# First discovery of the genus *Enicopus* Stephens (Coleoptera, Dasytidae) from China

**DOI:** 10.3897/BDJ.13.e144281

**Published:** 2025-01-28

**Authors:** Jialin Miao, Haoyu Liu, Junbo Tong, Xingke Yang, Yuxia Yang

**Affiliations:** 1 Key Laboratory of Zoological Systematics and Application, School of Life Sciences, Hebei University, Baoding, China Key Laboratory of Zoological Systematics and Application, School of Life Sciences, Hebei University Baoding China; 2 Hebei Basic Science Center for Biotic Interaction, Hebei University, Baoding, China Hebei Basic Science Center for Biotic Interaction, Hebei University Baoding China; 3 Key Laboratory of Zoological Systematics and Evolution, Institute of Zoology, Chinese Academy of Sciences, Beijing 100101, China, Beijing, China Key Laboratory of Zoological Systematics and Evolution, Institute of Zoology, Chinese Academy of Sciences, Beijing 100101, China Beijing China

**Keywords:** New faunistic record, alpha taxonomy, China, Cleroidea, Dasytidae

## Abstract

**Background:**

*Enicopus* Stephens, 1830, is classified within the subfamily Dasytinae of the family Dasytidae (Coleoptera, Cleroidea). Currently, it comprises two subgenera and 27 known species. This genus primarily inhabits south-central Europe, with certain species extending into west Asia; however, it has not been reported in China until now.

**New information:**

The genus *Enicopus* Stephens, 1830 is reported for the first time from China, following the discovery of E. (Enicopus) ater (Fabricius, 1787) in Xinjiang Autonomous Region. This species is thoroughly re-described and illustrated, including detailed depictions of the ultimate abdominal tergites and sternites, genitalia of both sexes, as well as tarsomeres 1 from the right front and hind legs of male.

## Introduction

The genus *Enicopus* was first proposed by Stephens 1830, with *Lagriaatra* Fabricius, 1787 designated as the type species. This genus is classified within the subfamily Dasytinae of the beetle family Dasytidae (Bahillo de la Puebla and López-Colón 2004). Adult specimens can be readily distinguished from other dasytid beetles by their unique tarsal appendages found in males (Liberti and Constantin 2009). *Enicopus* comprises two subgenera (Bahillo de la Puebla and López-Colón 2020), namely E. (Enicopus) Stephens, 1830 and E. (Parahenicopus) Portevin, 1931. These subgenera are differentiated, based on the characteristics of male tarsi. Specifically, tarsomeres 1 exhibit appendages on both front and hind tarsi in E. (Enicopus), whereas such appendages are present only on the hind tarsi in E. (Parahenicopus).

So far, it has been reported that there are 27 species (Bahillo de la Puebla and López-Colón 2020). Most of the species are distributed in south-central Europe, with one species spreading to Central Asia (Liberti and Constantin 2009). Amongst them, E. (Enicopus) ater (Fabricius, 1787) is the most widely distributed, extending from Western Europe to Central Asia (Liberti and Constantin 2009). It has been predicted to spread eastwards throughout Mongolia (Liberti and Constantin 2009), but has never been reported in China.

In this study, some materials of E. (Enicopus) ater were discovered in Xinjiang Autonomous Region, northwest China. They have been deposited in the Institute of Zoology, Chinese Academy of Sciences, Beijing since 1960. Due to the insufficient study of this beetle group in the Chinese fauna, they have been overlooked for a long time. Therefore, it is necessary to re-describe and illustrate it here to enhance its recognition and also enrich the beetle diversity of China.

## Materials and methods

In this study, we adhere to the conventional taxonomic classification of dasytid beetles as a separate family, Dasytidae (Majer 1994, Majer 2002, Mayor 2007, Bocakova et al. 2012, Constantin 2021), rather than regarding them as a subfamily within Melyridae (Constantin and Bahillo de la Puebla 2019, Gimmel and Mayor 2019, Gimmel and Mayor 2024). This study primarily focuses on the species descriptions of *Enicopus*, despite the ongoing debates regarding its higher classification, which falls outside the scope of this research. The specimens examined in this study are deposited in the Institute of Zoology, Chinese Academy of Sciences, Beijing, China (IZAS).

The specimens were initially soaked in water for softening, followed by the separation of their abdomens. The separated abdomens were then immersed in a 10% sodium hydroxide (NaOH) solution and heated at a constant temperature for several minutes using a metal bath. Once the fat had dissolved, they were transferred to a Nikon SMZ1500 stereomicroscope for the dissection of the pygidium, ultimate abdominal ventrite and genitalia. To facilitate observation, the spiculum gastrale, tegmen and median lobe were isolated respectively. The ovipositor was stained with haematoxylin. Subsequently, the dissected genitalia were placed on a glass slide with glycerol and photographed using a Leica M205A stereomicroscope before being stored in glycerol for preservation. A Canon EOS 80D digital camera was used to capture images of habitus which were later processed using Helicon Focus 7 software. Adobe Photoshop CC 2019 version 20.0.4 was utilised for editing in plate preparation. The body length was measured from the anterior margin of the head to the elytral apices and the width at the humeri. The terminology of genital segments follows Lawrence et al. (2010) and that of genitalia follows Gimmel and Mayor (2019).

## Taxon treatments

### Enicopus

Stephens, 1830

4B5B354C-F844-5EE8-8FD5-F39B321663E4

Enicopus (Enicopus) Stephens, 1830 - Stephens 1830: 318.
Enicopus (Parahenicopus) Portevin, 1931 - Portevin 1931: 453.
Lagria ater Fabricius, 1787

#### Diagnosis

Body black and covered with long hairs. Pronotum with two sinuous lateral grooves on disc. Front tibiae terminating in a short hooked appendage, tarsi with at least one pair of spines or appendages on tarsomeres 1 in males, while simple in female, all tarsal claws fitted with a membrane almost as long as the claws themselves. Tegmen with two well-developed and symmetrical lobes at apex, densely covered with long hairs; internal sac of median lobe with more than two pairs of large spines at apical part.

#### Distribution

China (new record: Xinjiang), Spain, Portugal, Albania, Algeria, Armenia, Austria, Azerbaijan, Bosnia-Herzegovina, Bulgaria, Caucasus, Croatia, Czechia, France, Germany, Georgia, Greece, Hungary, Iran, Italy, Kazakhstan, Kyrgyzstan, Macedonia, Mongolia, Montenegro, Romania, Russia, Serbia, Slovakia, Sweden, Switzerland, Turkey, Ukraine, Uzbekistan.

### Enicopus (Enicopus) ater

(Fabricius, 1787)

6924A9D6-15C2-560B-AB37-AC69B3A6535D

Lagria atra Fabricius, 1787 - Fabricius 1787: 94.
Melyris ater (Fabricius, 1787) - Olivier 1790: 9; Olivier 1808: plate 2, figs. 8a-8e.
Dasytes ater (Fabricius, 1787) - Germar 1817: 209; Stephens 1829: 136; Küster 1849: 15.
Enicopus ater (Fabricius, 1787) - Stephens 1830: 318; Stephens 1833: 46; Stephens 1839: 195; Spry and Shuckard 1840: 75; Mayor 2007: 407 (syn. of *E.pilosus* Scopoli, 1763); Liberti and Constantin 2009: 298.

#### Materials

- **Type status:** Other material. **Occurrence:** recordedBy: Shuyong Wang; individualCount: 8; sex: 4 males, 4 females; lifeStage: adult; occurrenceID: 88CA14BD-017B-5FDF-8245-6F0B22E842FE; **Location:** country: China; stateProvince: Xinjiang; county: Qinghe; verbatimElevation: 1450 m; **Event:** year: 1960; month: 7; day: 3; **Record Level:** institutionID: Institute of Zoology, Chinese Academy of Sciences; institutionCode: IZAS

#### Description

**Figure 1.**
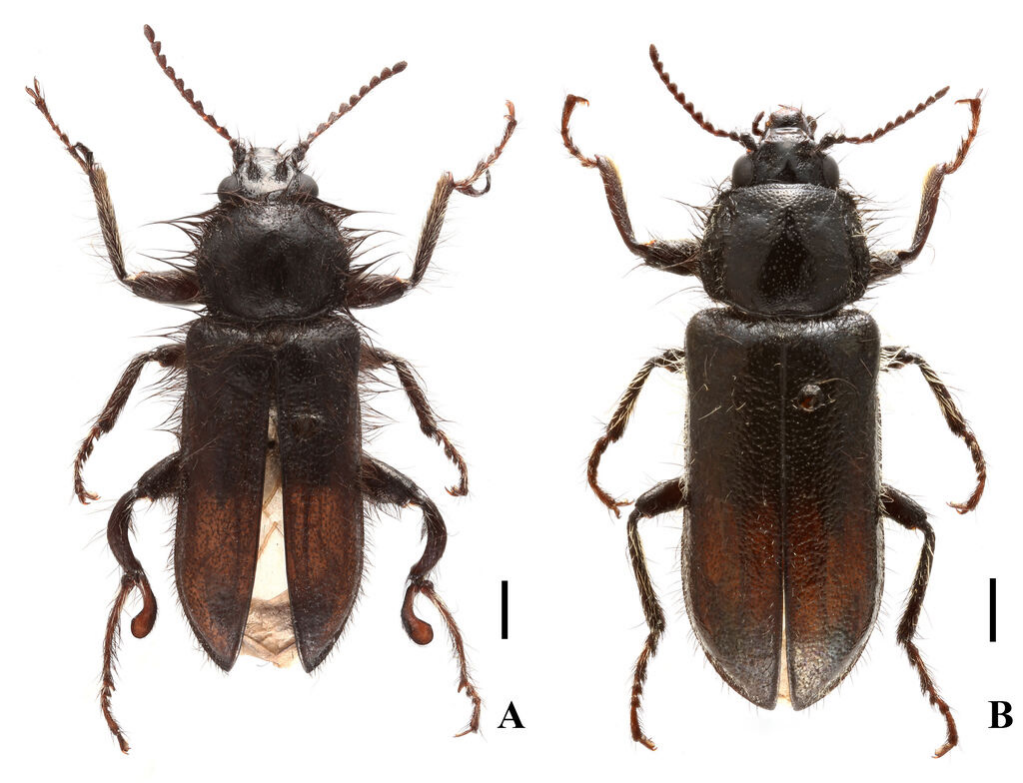
Habitus of Enicopus (Enicopus) ater (Fabricius, 1787), dorsal view: **A** male; **B** female. Scale bars: 1.0 mm.

**Male** (Fig. 1A). Body length 7.4–9.4 mm, width 2.3–2.9 mm.

Body black with lustre, except for antennomeres 3 and 4 yellow at apices. Body densely and coarsely punctate on surface, as well as covered with short and recumbent black pubescence, grey on antennomeres 3–11, tibiae and tarsi. Vertex behind eyes densely covered with long erect black hairs. Antennomeres 1 and 2 covered with a few erect black hairs. Pronotum densely covered with long erect black hairs around all margins, of which that on lateral margins longer than those on anterior and posterior margins. Elytra covered with long erect black hairs along margins, which are progressively shortened towards apices.

Head width across eyes narrower than anterior margin of pronotum, present with a pair of shallow depressions on vertex. Antennae quite short, extending to posterior margin of pronotum when inclined, antennomeres 1 nearly ellipsoid, 1.5 times as long as wide, 2 short, apical half expanded, 3–10 triangular with truncate apex, 11 elongate and fusiform, twice as long as wide. Ultimate maxillary palpomere fusiform, about 1.7 times longer than the penultimate one.

Pronotum transverse and 1.3 times as wide as long, widest near middle, anterior margin slightly arcuate, lateral margins slightly diverging posteriorly and regularly rounded with distinct edges, posterior margin slightly bisinuate.

Elytra elongate and parallel-sided, 2.1–2.3 times longer than wide at humeri, 2.3–2.5 times longer than pronotum, rounded at humeri, acute at apices.

**Figure 2.**
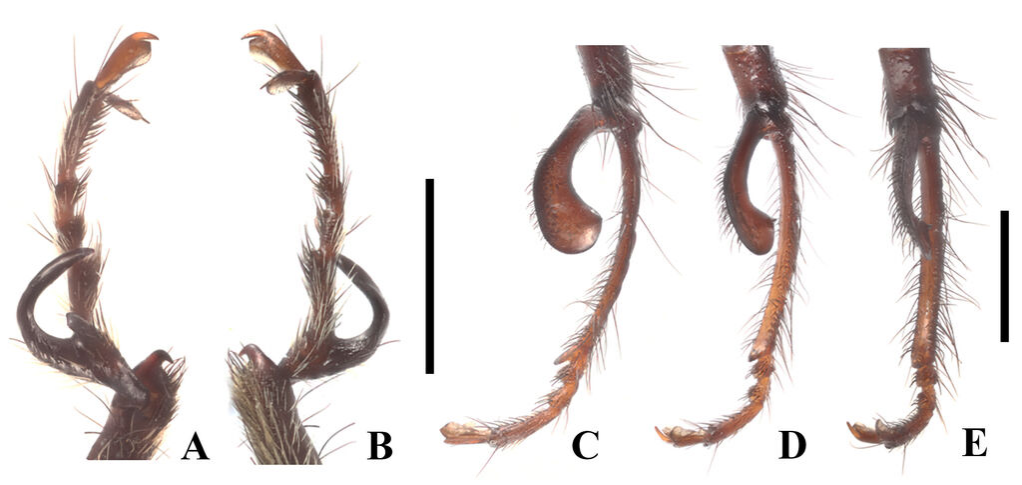
Enicopus (Enicopus) ater (Fabricius, 1787), male: **A** front tarsi, ventral view; **B** front tarsi, dorsal view; **C** hind tarsi, dorsal view; **D** hind tarsi, dorso-lateral view; **E** hind tarsi, lateral view. Scale bars: 1.0 mm.

Front femora slightly swollen, each tibia with a pair of small and sharply-hooked spurs at apex, tarsomeres 1 with a large and sharply hooked appendage on outer side, which bear a large gable-shaped tooth on inner base (Fig. 2A–B); middle tarsomeres 1 short, with a pair of small sharp teeth; hind femora slightly swollen, tibiae slightly swollen, distinctly curved, tarsomeres 1 with a large spoon-shaped appendage on outer base, which is bent inwards and slightly wider at apical part than basal part, external angle acute and pointing upwards, tarsomeres 2 very long and twice longer than combined length of tarsomeres 3–5 and terminating into a small tooth at apex; tarsal claws symmetrical, with a pair of broad membranous appendages, which slightly shorter than the claws (Fig. 2C–E).

**Figure 3.**
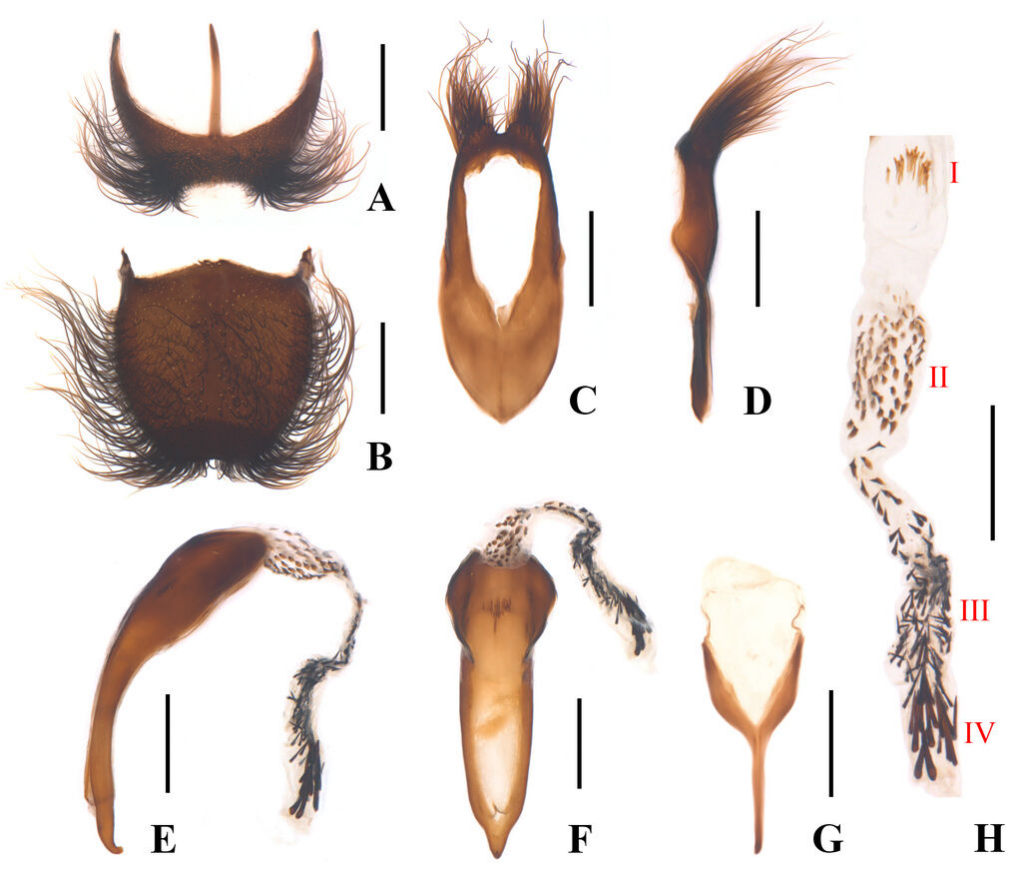
Enicopus (Enicopus) ater (Fabricius, 1787), male: **A** ultimate abdominal ventrite (apical sternite), ventral view; **B** pygidium (apical tergite), dorsal view; **C** tegmen, ventral view; **D** tegmen, lateral view; **E** median lobe, lateral view; **F** median lobe, ventral view; **G** spiculum gastrale, ventral view; **H** internal sac; **I–IV** the four types of spines covered on the internal sac. Scale bars: 0.5 mm.

Ultimate abdominal ventrite (Fig. 3A) saddle-form, narrowed posteriorly, 1.2–1.3 times as wide as long, shallowly and trapezoidally emarginate in middle of posterior margin, present with a slender central process at anterior margin, which does not extend over antero-lateral angles, surface densely covered with long black hairs along lateral margins. Pygidium (Fig. 3B) shield-form, 1.0–1.1 times longer than wide, feebly narrowed posteriorly, hardly emarginate in middle of posterior margin, largely and triangularly protuberant in middle of anterior margin, with antero-lateral angles obviously protruding, surface covered with long black hairs in centre and along lateral margins. Aedeagus: tegmen (Fig. 3C and D) nearly bullet-shaped, present with a pair of symmetrical and nearly semicircular lobes at apex, wider than long, where it is densely covered with long hairs; median lobe strongly bent ventrally, dorso-ventrally flattened apically in lateral view (Fig. 3E), with apex slightly hooked ventrally, slightly elongated at apex in ventral view (Fig. 3F); internal sac (Fig. 3H) long and membranous, fitted with four types of spines: the first type (I) located at base, a dozen or so, yellow and slender, arranged in parallel, the second type (II) dozens, brown, short and nearly triangular, the third type (III) dozens, black and slender and the fourth type (IV) located at the apical part, a dozen or so, black, stout and long. Spiculum gastrale Y-shaped (Fig. 3G).

**Figure 4.**
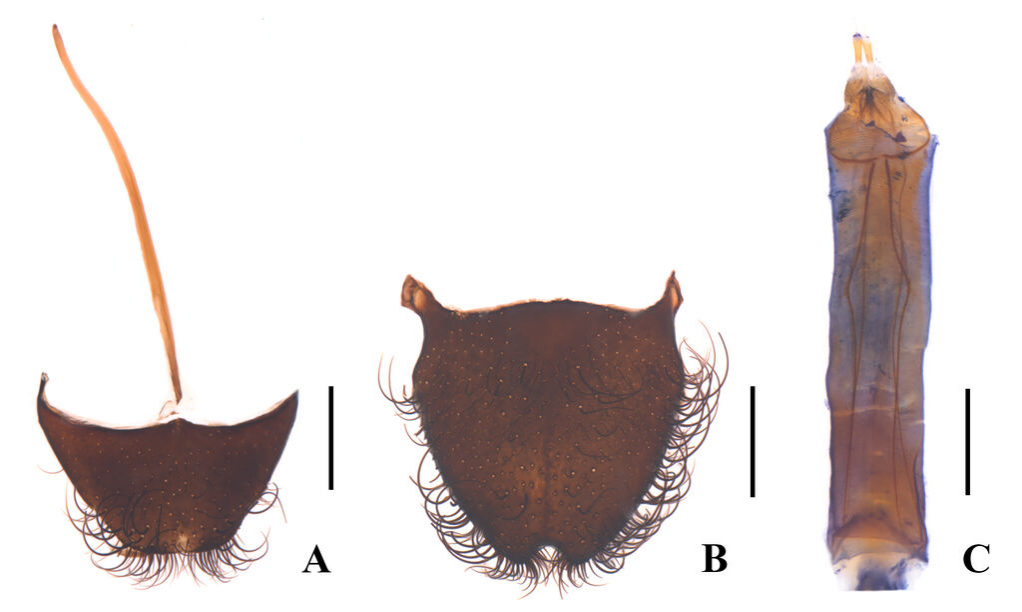
Enicopus (Enicopus) ater (Fabricius, 1787), female: **A** ultimate abdominal ventrite (apical sternite), ventral view; **B** pygidium (apical tergite), dorsal view; **C** ovipositor, ventral view. Scale bars: 0.5 mm.

**Female** (Fig. 1B). Similar to male, but body larger, length 8.7–9.6 mm, width 3.1–3.4 mm. Eyes less prominent. Front tarsomeres 1 without any appendage; middle tarsi simple; hind tibiae normally slender, straight, tarsomeres 1 without any appendage, tarsomeres 2 much shorter than that of male. Ultimate abdominal ventrite (Fig. 4A) trapezoidal, strongly narrowed posteriorly, posterior margin nearly truncate, present with a very long central process at anterior margin, which distinctly extends over postero-lateral angles. Pygidium (Fig. 4B) strongly narrowed posteriorly, semicircularly emarginate in middle of posterior margin, arcuate at anterior margin, antero-lateral angles obviously protruding. Ovipositor (Fig. 4C) slender and membranous, gonostylus feebly long and nearly cylindrical.

#### Diagnosis

This species can be distinguished from all other species of E. (Enicopus) by the characteristics of tarsal appendages and aedeagus. It looks more similar to E. (Enicopus) pilosus (Scopoli, 1763), but can be distinguished by the combination of the following characters: appendage on hind tarsomeres 1 of male flattened and spoon-shaped, with the external angle pointing upwards; median lobe strongly curved ventrally in lateral view; tegmen with a pair of nearly semicircular lobes at apex, which are separated and feebly wider than long. Unlike in *E.pilosus*, appendage on hind tarsomeres 1 of male is sickle-shaped, with apical part bent upwards (Liberti and Constantin 2009: fig. 23); median lobe is slightly curved dorsally in lateral view (Liberti and Constantin 2009: fig. 8); tegmen bears a pair of slender and parallel lobes at apex, which are much longer than wide (Liberti and Constantin 2009: fig. 12).

#### Distribution

China (new record: Xinjiang), Albania, Armenia, Austria, Azerbaijan, Bosnia-Herzegovina, Bulgaria, Caucasus, Croatia, southern France, Georgia, Greece, Hungary, Iran, Italy, Kazakhstan, Kyrgyzstan, Macedonia, Montenegro, Romania, Russia (incl. South Siberia), Serbia, Turkey, Ukraine, Uzbekistan.

#### Notes

Liberti and Constantin (2009) presented a detailed list of all synonyms for this species, which are omitted here. Meanwhile, they depicted the internal sac of the median lobe with 3-5 spines at the apex (Liberti and Constantin 2009: fig. 11), but there are many more shown in our material (Fig. 3H). Later, they provided an illustration of the adult male, whose hind tarsomeres 2 is slightly longer than the combined length of the last three tarsomeres (Constantin and Liberti 2011: fig. 27), while it is much longer in our material (Fig. 1A). Nevertheless, we identify them as E. (Enicopus) ater because more or less variations can occur in such a widespread species.

## Supplementary Material

XML Treatment for Enicopus

XML Treatment for Enicopus (Enicopus) ater
